# Cyclophilin A protects mice against infection by influenza A virus

**DOI:** 10.1038/srep28978

**Published:** 2016-06-29

**Authors:** Jing Li, Can Chen, Gary Wong, Wei Dong, Weinan Zheng, Yun Li, Lei Sun, Lianfeng Zhang, George F. Gao, Yuhai Bi, Wenjun Liu

**Affiliations:** 1 CAS Key Laboratory of Pathogenic Microbiology and Immunology, Institute of Microbiology, Chinese Academy of Sciences, Beijing 100101, China; 2 Key Laboratory of Human Disease Comparative Medicine, Ministry of Health, Institute of Laboratory Animal Science, Chinese Academy of Medical Sciences & Comparative Medical Center, Peking Union Medical College, Beijing, 100021 China; 3 Center for Influenza Research and Early-warning (CASCIRE), Chinese Academy of Sciences, Beijing 100101, China

## Abstract

Our previous studies indicate that Cyclophilin A (CypA) impairs the replication of influenza A virus *in vitro*. To further evaluate the antiviral functions of CypA and explore its mechanism, transgenic mice with overexpression of CypA by two specific promoters with SPC (CypA-SPC) or CMV (CypA-CMV) were developed. After challenge with the A/WSN/33(H1N1) influenza virus, CypA-SPC and CypA-CMV transgenic mice displayed nearly 2.5- and 3.8-fold stronger disease resistance to virus infection, respectively, compared to wild-type animals. Virus replication, pathological lesions and inflammatory cytokines were substantially reduced in both lines of transgenic mice. In addition, after infection there was an upregulation of genes associated with cell migration, immune function, and organ development; and a downregulation of genes associated with the positive regulation of immune cells and apoptosis in the peritoneal macrophages of CypA-overexpressing transgenic mice (CypA+). These results indicate that CypA is a key modulator of influenza virus resistance in mice, and that CypA+ mice constitutes an important model to study the roles of CypA in the regulation of immune responses and infections.

Cyclophilin A (CypA) is a typical member of the cyclophilin family, which exhibits peptidyl-prolyl cis-trans isomerase (PPIase) activity. CypA is distributed ubiquitously in mammalian and avian tissues^1^,^2^,^3^, displays a chaperon-like activity, and takes part in protein-folding processes^4^,^5^,^6^. It is primarily found in the cytoplasm and can be secreted into the extracellular environment^1^.

CypA plays an important role in regulating immune responses^7^. It is the primary mediator of immunosuppression by cyclosporine (CsA)^8^, which is widely used in humans to prevent organ transplant rejection. CsA bind to CypA in its hydrophobic pocket, inhibiting PPIase activity. However, the inhibition of CypA enzymatic activity is not responsible for immunosuppressive pharmacological effects^7^. Rather, the CsA-CypA complex binds to and inhibits calcineurin, a calcium-activated serine/threonine-phosphatase^9^,^10^, which is important for T-cell activation through the nuclear import of nuclear factor of activated T-cells (NF-AT) transcription factors. Hence, T-cell activation is blocked in the presence of the CsA-CypA complex, resulting in the reduced expression of pro-inflammatory cytokines and an overall decrease in the immune response^8^.

CypA-knockout mice have been shown to develop a spontaneous Type I hypersensitive response, with elevated levels of serum IgG1 and IgE, as well as tissue infiltration by mast cells and eosinophils which are driven by a dysregulated Th2 response^11^. CypA suppresses the development of CD4+ T-cell responses through the inhibition of Interleukin-2 tyrosine kinase (Itk)^11^ by binding to the SH2 domain of Itk^7^,^11^,^12^. Furthermore, CypA is a mediator of pro-inflammatory responses and a potent chemoattractant for human monocytes, neutrophils, eosinophils, and T-cells^13^. CD147 was identified as the main signaling receptor for CypA, and these two molecules contribute to the recruitment of neutrophils into the lung tissues of mice after they are given an intranasal (IN) dose of lipopolysaccharides^14^. Recent studies show that CypA promotes the nuclear translocation of NF-κB/p65 and stimulates NF-κB phosphorylation and activation, resulting in enhanced NF-κB activity and the altered expression of its target genes^15^,^16^. CypA also interacts with apoptosis-inducing factor to promote chromatinolysis^17^ and plays a role in the progression of some diseases, including peripheral artery disease, chronic kidney disease, and multiple myeloma^14^,^18^,^19^,^20^.

CypA also directly incorporates itself into several virus particles, such as human immunodeficiency virus type 1 (HIV-1)^21^, influenza virus^22^,^23^, vaccinia virus (VV)^24^, and vesicular stomatitis virus (VSV)^25^. Moreover, CypA plays a critical role in the successful infectivity and replication of HIV-1, HCV, HBV, and VSV, as well as the protozoan parasite *Leishmania major*^5^,^25^,^26^,^27^,^28^,^29^,^30^. However, CypA suppresses the replication of rotavirus^31^, infectious bursal disease virus^32^, tomato bushy stunt tombusvirus (TBSV)^33^, mouse cytomegalovirus^34^, and influenza virus^1^,^26^.

Therefore, CypA can play both a beneficial or detrimental role in regulating the balance between the host and a pathogen, and the role depends on individual conditions that should be further confirmed in animal models. In our previous studies, we found that CypA inhibits influenza virus replication *in vitro*^22^,^35^,^36^. To investigate whether CypA is able to suppress virus replication *in vivo*, we developed transgenic mice over-expressing CypA via a specific promoter in the lungs (CypA-SPC) or all over the body (CypA-CMV). The different mice genotypes were characterized for their susceptibility to influenza virus after a challenge with A/WSN/33 (H1N1). Weight change, as well as virus titers, histopathology, and immunohistochemistry of the lungs were examined after challenge. Additionally, cytokine responses and an analysis of transcriptomes from the peritoneal macrophages of infected mice are presented herein.

## Results

### Development and characterization of transgenic mice over-expressing CypA

**Figure 1:**
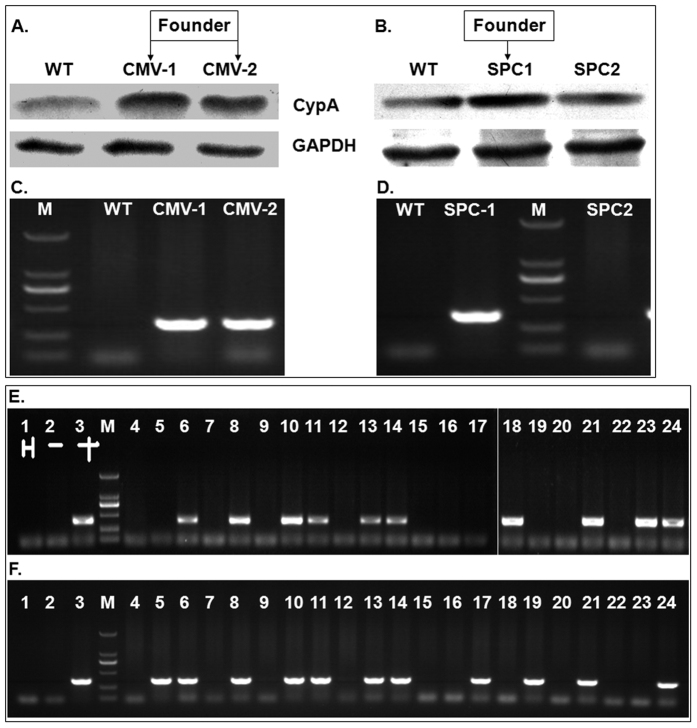
Construction and identification of transgenic mice over-expressing CypA. The founders of transgenic mice lines over-expressing CypA were identified at the protein level in lung tissue using western blots (**A,B**) and at the DNA level using PCR for the specific CypA-expression promoter in the genome (**C,D**). CMV and SPC represent the body and lung over-expression mice, respectively. The first filial generations were identified with PCR for the specific CypA-expression CMV (**E**) and SPC (**F**) promoters in the genome. M represents the DNA marker (from bottom to top: 100, 250, 500, 750, 1000 and 2000 bp) in panels C–F.

Transgenic C57BL/6 mice over-expressing CypA via the SPC or CMV promoter were generated by the microinjection method. The founders of the CypA+ mouse lines were confirmed for CypA expression at the protein and the nucleic acid levels, with greater than twice the expression levels of CypA in the lungs compared to wild type mice (Fig. 1A,B), and possessing the specific promoter for CypA expression in tail tissues (Fig. 1C,D). The first filial generation, produced by the female CypA+ and male wild type mice, were identified by PCR for the presence of the specific promoter (Fig. 1E,F). The rates of progeny mice testing positive for CypA-CMV and CypA-SPC were 53.1% and 47.4%, respectively.

### Susceptibility of CypA+ mice to influenza virus infections

**Figure 2:**
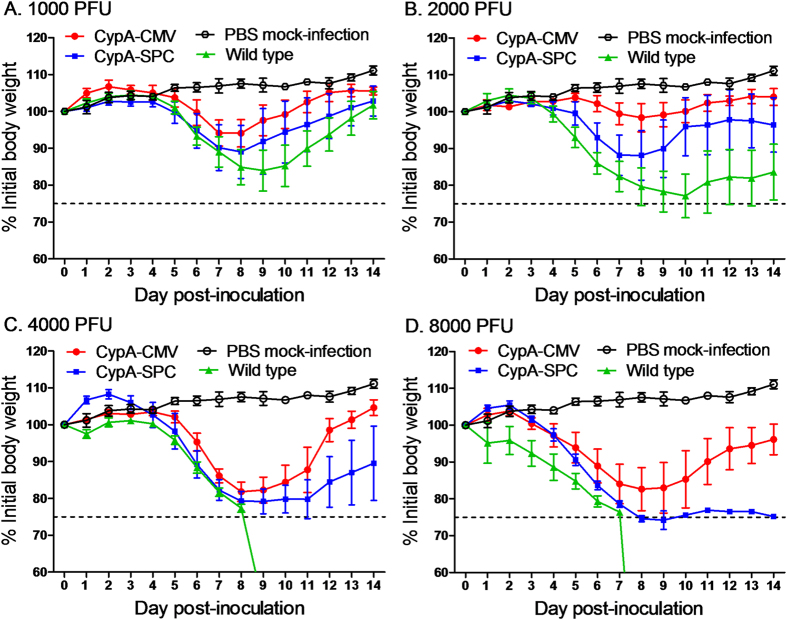
The antiviral activity of CypA transgenic mice, as evaluated by influenza infection at various doses. Wild type, CypA-CMV, and -SPC over-expressing C57BL/6 mice were inoculated i.n. with WSN at doses of 1000 (**A**), 2000 (**B**), 4000 (**C**), and 8000 PFU (**D**), respectively (n = 5 per dilution, 20 mice per group). The mice in the control group were i.n. inoculated with PBS for mock infection. The body weights of five mice in each group were monitored daily for 14 days and are expressed as a percentage of the initial value. The data represents the mean of five mice in each group.

To investigate the susceptibility of CypA+ mice to infection with influenza A virus, the 50% median lethal dose (MLD_50_) was determined for each genotype by challenge with a serial dilution series of A/WSN/1933 (H1N1) virus at a dose of 1000, 2000, 4000, or 8000 plaque forming units (PFUs) (n = 5 per dilution, 20 mice per group). At each challenge dose, the wild type C57BL/6 mice showed more severe clinical symptoms after infection, including weight loss, ruffled fur, and inactivity, compared to the CypA+ mice. Interestingly, the CypA-SPC mice showed slightly more weight loss than the CypA-CMV animals (Fig. 2). The MLD_50_ values of A/WSN/33(H1N1) against wild type, CypA-SPC, and CypA-CMV mice were calculated to be 1561, 3892, and 5907 PFU, respectively. Therefore, the CypA-SPC and CypA-CMV mice were 2.5- and 3.8-fold more resistant, respectively, against infection with influenza A virus (Table 1). These results show that overexpression of CypA in the mouse contributed to disease resistance in the context of infection with influenza A virus.

### Clinical symptoms of mice infected by A/WSN/33(H1N1)

**Figure 3:**
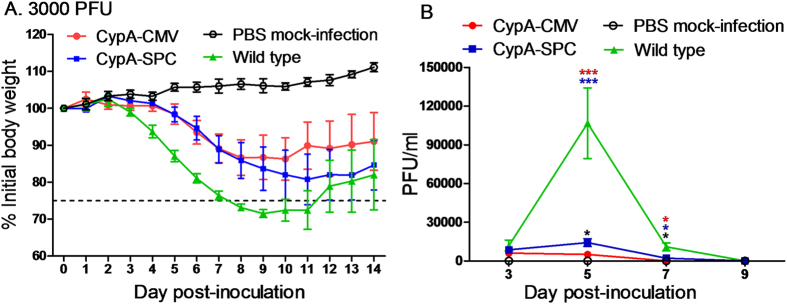
Body weight changes and lung virus titers of the mice infected with 3000 PFU A/WSN/33(H1N1) virus. The wild type, CypA-CMV, and CypA-SPC mice were inoculated i.n. with A/WSN/33(H1N1) virus at a dose of 3000 PFU. The body weights of five mice in each group were monitored daily for a 14-day observation period and are expressed as a percentage of the initial value (**A**). Three mice per group were euthanatized at 3, 5, 7, 9, 11, and 14 d.p.i., and the LVTs were titrated in MDCK cells by standard plaque assay to determine viral titers (**B**). The LVTs were compared with a paired-sample *t-*test and two-way ANOVA, respectively, *P < 0.05, **P < 0.01, and ***P < 0.001. The black, blue, and red asterisks represent the results compared to wild type, CypA-SPC, and CypA-CMV mice, respectively.

To compare the clinical symptoms of mice after infection with a single challenge dose, wild type, CypA-SPC, and CypA-CMV mice were infected with 3000 PFU of A/WSN/33(H1N1). This infection dose was chosen on the basis that difference in clinical symptoms is the most easily distinguishable amongst the different mouse genotypes. Clinical signs were monitored over a 14-day period. After challenge, the wild type mice displayed more severe clinical signs with apparent inactivity, ruffled fur, decreased food intake, and higher weight loss than the CypA+ mice. The weights of the wild type mice begin to decrease at 3 d.p.i., losing up to 28–29% of initial weight, before gradually recovering. In contrast, the CypA+ mice began to lose weight around 5 d.p.i., and the weight loss was significantly lower than that of the wild type mice (Fig. 3A). The CypA-SPC and CypA-CMV mice lost approximately 20% and 14% of their initial weights, respectively.

### Virus replication in CypA+ and wild type mice

The level of virus replication observed after infection is correlated to the susceptibility of the host to the pathogen. Therefore, the lung virus titers (LVTs) of the infected mice groups were tested after A/WSN/33(H1N1) infection at a challenge dose of 3000 PFU. We found that the virus was detected in the wild type, CypA-SPC, and CypA-CMV groups at 3 d.p.i. with LVTs of ~12000, ~8700, and ~6000 PFU/ml, respectively. At 5 d.p.i., the LVTs increased to ~100000, ~14000, and ~5000 PFU/mL in the wild type, CypA-SPC, and CypA-CMV mice, respectively (Fig. 3B). At 7 d.p.i., the LVTs dropped to ~10000 and ~2000 PFU/mL in the wild type and CypA-SPC mice, respectively, and were undetectable in the CypA-CMV animals. The virus was also eliminated in the wild type and CypA-SPC mice by 9 d.p.i (Fig. 3B). These results show that the virus was suppressed in CypA+ mice compared to wild type. Moreover, the CypA-CMV mice displayed better levels of influenza virus inhibition than the CypA-SPC mice.

### Pathological changes in CypA+ and wild type mice

**Figure 4:**
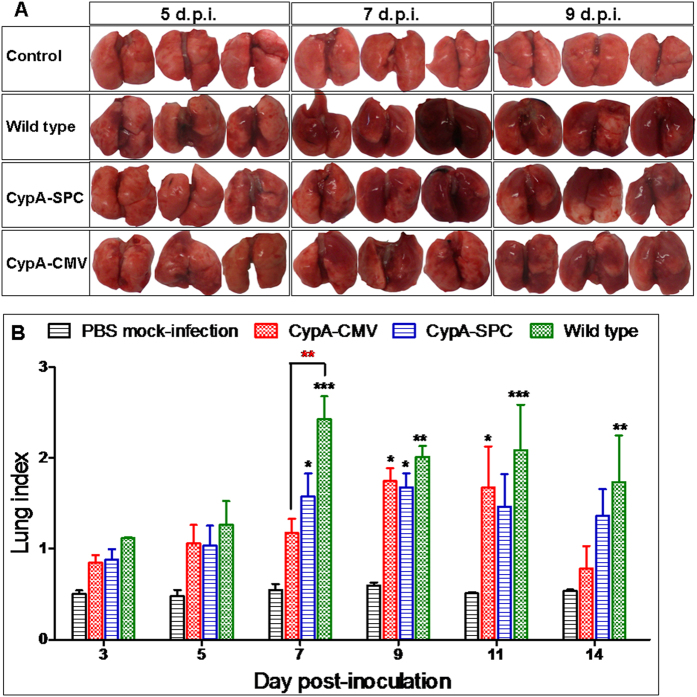
Gross pathology of the lungs in mice infected with A/WSN/33(H1N1) virus. The mice in each group were inoculated i.n. with 3000 PFU WSN. Three mice each from the PBS mock-infection control group, wild type, CypA-CMV, and CypA-SPC C57BL/6 mice were euthanatized at 3, 5, 7, 9, 11 and 14 d.p.i., the gross pathology of the infected lungs were observed, and photographs of the lungs at 5, 7, and 9 d.p.i. are shown in (**A**). The lung indices (**B**) were compared to each other by two-way ANOVA; *P < 0.05, **P < 0.01, and ***P < 0.001. The black and red asterisks represent the results compared to control group and the wild type mice, respectively.

The pathologic changes in the lungs of each experimental group were examined after a challenge dose of 3000 PFU. Three mice from each group were euthanized at time points of 3, 5, 7, and 9 d.p.i. and the lungs were harvested. Gross pathology with engorgement was observed at 3 d.p.i. Significant engorgement emerged at 5 d.p.i. and was most severe at 7–9 d.p.i. before gradually recovering. Notably, the wild type mice displayed more severe gross pathology than their CypA+ counterparts during the course of the infection (Fig. 4A). Consistent with the gross pathologic changes, the lung indices of the infected wild type mice were consistently higher than that of the CypA+ mice, but the difference was not always statistically significant (Fig. 4B).

**Figure 5:**
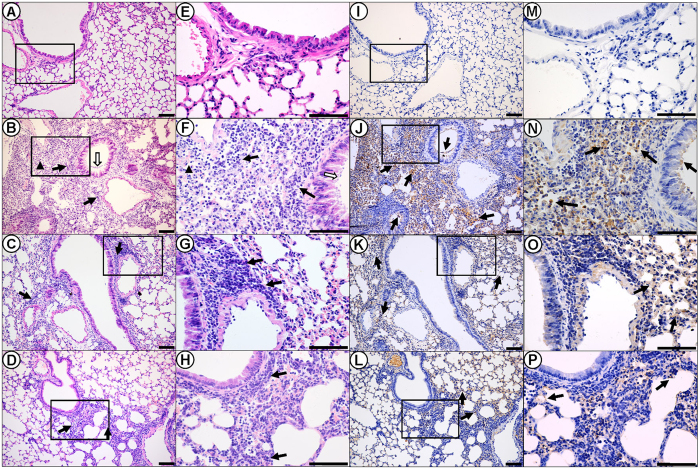
Histopathoglogical and immunohistochemical finding in the lungs of infected mice at 5 d.p.i. Lung histopathology sections (magnification, 200×) of mice were shown at 5 d.p.i. for the (**A**) PBS mock-infection control, (**B**) wild type, (**C**) CypA-SPC, and (**D**) CypA-CMV groups. (**E–H**) Enlargements (600×) for panels (**A–D**), respectively. Inflammatory cell infiltration, deciduous epithelium mucosae and inflammatory cells in the bronchial lumen, as well as hemorrhage are denoted with thick black arrows, thick white arrows, and black triangles, respectively. (**I–L**) Immunohistochemically stained sections (200×) corresponding to the histopathology sections, respectively. (**M–P**) Enlargements (600×) for panels I to L, respectively. Positive signals are denoted with thick solid arrows. Scale bar = 100 μm.

H&E staining was performed to evaluate the histopathological changes in mouse lungs after challenge. Severe bronchopneumonia was observed 5 d.p.i. (Fig. 5A–H), and severe interstitial pneumonia occurred at ~7 d.p.i. in the wild-type mice (Fig. S1). In contrast, the CypA+ mice showed comparatively less histopathological changes after infection, from mild to moderate/severe bronchopneumonia. The inflammatory cells also infiltrated in higher numbers in the lungs of the infected wild type mice compared to CypA+ mice (Fig. 5A–H and Fig. S1). In accordance to the histopathological changes, the virus infected cells stained by M1 protein in the lungs of CypA+ mice was significant less than that of wild type (Fig. 5I–P).

### Cytokine responses in CypA+ and wild type mice

**Figure 6:**
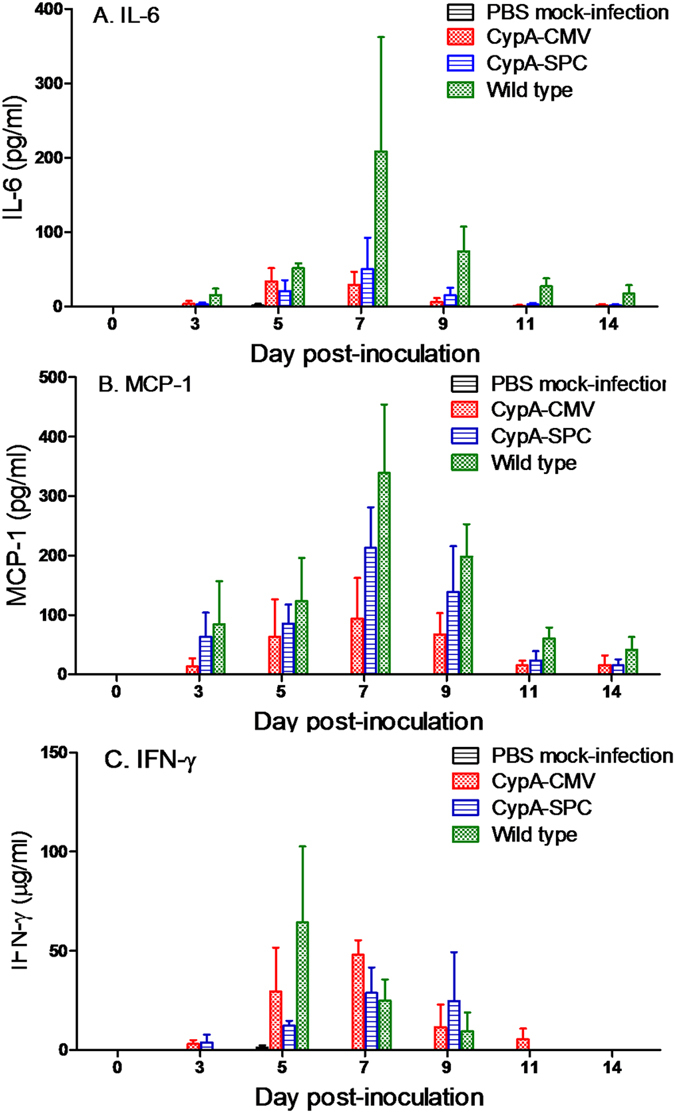
Secretion of cytokines in mice after infection with A/WSN/33(H1N1) virus. Three mice per group were infected with 3000 PFU A/WSN/33(H1N1), euthanatized at 3, 5, 7, 9, 11, 14 d.p.i., and the sera were extracted for cytokine detection

Cytokine levels in the sera of the infected mice were then tested to investigate the antiviral mechanisms of CypA. After infection with A/WSN/33(H1N1), IL-6 and MCP-1 levels began to rise at 3 d.p.i. and peaked around 7 d.p.i. before dropping at 9 d.p.i. in both the CypA+ and wild type mice. IL-6 and MCP-1 levels in the wild type mice were consistently higher than in CypA+ mice throughout the infection (Fig. 6A,B). In contrast, IFN-γ levels were consistently higher for CypA+ mice than their wild type counterparts (Fig. 6C).

### Transcriptomics of peritoneal macrophages from infected CypA+ and wild type mice

**Figure 7:**
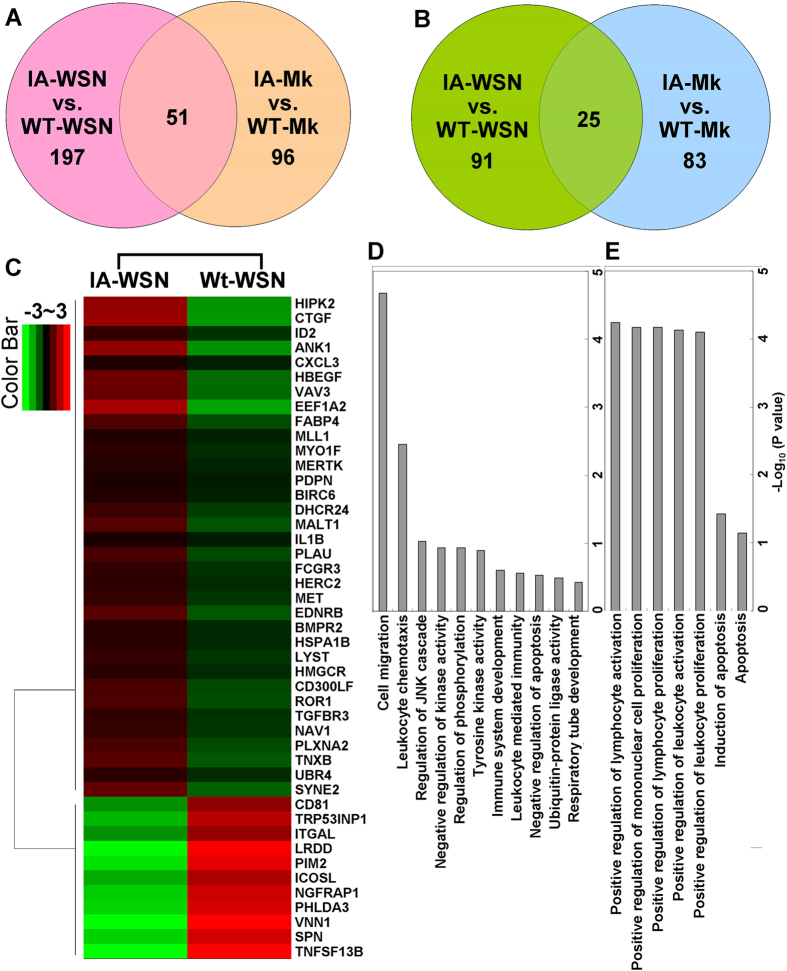
Transcriptomics analysis of the peritoneal macrophages from mice infected by A/WSN/33(H1N1) virus. Peritoneal macrophages from the wild type and CypA-CMV transgenic C57BL/6 mice were infected with A/WSN/1933(H1N1) virus (MOI = 2). After 8 h.p.i., the total RNA of the infected and control cells were extracted and analyzed by RNA-seq. Up- (**A**) and down-regulated genes (**B**) from cells of CypA transgenic mice were used to generate Venn diagrams and the heat-map (**C**). The gene functional classification of the specific up- (**D**) and down-regulated genes (**E**) with the most significance (p < 0.05) were analyzed by DAVID Bioinformatics Resources.

Macrophages perform critical roles in both innate and adaptive immunity and are distributed throughout the host in every organ type. Thus, macrophages act as an important immunologic barrier for the host defense system against infection and injury^37^. A recent study shows that peritoneal macrophages appear to be more mature than bone marrow-derived macrophages and splenic macrophages^38^. Based on these considerations, peritoneal macrophages were used to study the potential immune mechanism of CypA. The transcriptomes of the peritoneal macrophages of wild type and CypA+ mice were investigated *ex vivo*. Eight hours after infection with the A/WSN/33(H1N1) virus at a multiplicity of infection (MOI) of 2, 197 specific up- and 91 down-regulated genes were identified in the infected peritoneal macrophages of CypA-CMV mice compared to wild type (Fig. 7A,B). The functions of these differently expressed genes (DEGs) were then analyzed by DAVID Bioinformatics Resources with the Gene Functional Classification method. The upregulated genes were associated with cell migration, immune function, and organ development (Fig. 7C). In contrast, the downregulated genes were associated with the positive regulation of immune cells and apoptosis (Fig. 7C). Interestingly, there were also 96 up- and 83 down-regulated DEGs in the uninfected peritoneal macrophages of CypA-CMV mice compared to wild type (Fig. 7A,B), and phenotypic differences between the two mouse lines as a result of these DEGs (ie. immune responses, organ development, etc.) will be an interesting topic of study in the future.

## Discussion

CypA is the most abundant intracellular protein in the cyclophilin family and plays important roles in regulating immune responses, involving T-cell activation, pro-inflammatory responses, and the innate immune system^7^,^9^,^10^,^11^,^15^,^16^. CypA expression is correlated with apoptosis and the development of some diseases^14^,^18^,^19^,^20^. In addition, CypA contributes to the infection and infectivity of some pathogens, such as HIV-1, HCV, HBV, VSV, and *L. major*^5^,^25^,^26^,^27^,^28^,^29^,^30^, though it also suppresses the replication of rotavirus^31^, infectious bursal disease virus, TBSV, mouse cytomegalovirus, and influenza virus^1^,^26^,^32^,^33^,^34^. However, nearly all of these data come from *in vitro* experiments and lack *in vivo* support from animal models. Hence, it is necessary to confirm these conclusions *in vivo*, which would help to further understand the functions of CypA. CypA+ transgenic mice would undoubtedly help to resolve these discrepancies.

In the present study, we found that CypA+ mice exhibit antiviral activity against influenza virus challenge, and that overexpression of CypA resulted in the upregulation of antiviral genes in the host, likely contributing to disease resistance. The lung indices, gross pathology, and histopathological changes we observed demonstrated that inflammatory responses were significantly inhibited in CypA+ mice after infection (Figs 4 and 5). Cytokine responses, including IL-6, MCP-1, IFN-γ, and TNF-α levels, are closely related to the host damage caused by the influenza virus infection in the host^39^,^40^,^41^,^42^,^43^,^44^,^45^. Interestingly, the serum IL-6 and MCP-1 levels were obviously repressed in the CypA+ mice, whereas changes in IFN-γ expression were milder and more prolonged than that of wild type mice (Fig. 6). However, there was no statistical significance for the cytokine concentrations between the CypA+ and wild type groups as determined by two-way ANOVA, which may be due to the limited numbers of animals (n = 3) at each detection timepoint. Our previous studies show that CypA inhibits influenza virus replication *in vitro* through the direct interaction between CypA and the viral M1 protein^22^,^35^. Hence, the antiviral function of CypA was thought to occur via two routes, involving the virus itself and the host.

IFN-γ secretion from CypA+ mice was found to be higher than their wild type counterparts after influenza virus infection. IFN-γ is produced by NK cells and specific subsets of T-lymphocytes (including CD4 Th1 and CD8 cytotoxic T-lymphocyte effector cells), is known to be critical for innate and adaptive immunity against viral, some bacterial, and protozoan infections^43^,^44^. IFN-γ acts as an immunostimulatory and immunomodulatory factor regulating immune responses in the CypA+ mice. Furthermore, IFN-γ is an important activator of macrophages and an inducer of Class I major histocompatibility complex (MHC) molecule expression^46^.

The host requires a robust immune response to suppress influenza virus infection^47^. Transcriptomic analyses of mouse peritoneal macrophages revealed that the upregulated genes associated with cell migration and leukocyte chemotaxis, which may increase the efficiency of immune cell transport between infected sites and other organs. Several leukocyte-mediated immunity-associated genes were also up-regulated (Fig. 7), which may increase the utilization rate for the immune cells. The up-regulation of genes in immune system development would be important in helping the host mount a robust inflammatory response, whereas up-regulation of genes related to respiratory tube development helps the host to repair tissue damage during the infection. In contrast, a down-regulation of genes associated with positive regulation of immune cell proliferation and activation decrease the chance of aberrant cytokine responses. Suppressed IL-6 and MCP-1, and prolonged IFN-γ levels may be related to changes in the levels/activities of host factors that are influenced by CypA in CypA+ mice. Furthermore, apoptosis plays an important role in tissue damage during influenza virus infections^46^. Therefore, the up-regulation of genes that down-regulate apoptosis delays the formation of pathological lesions. The up-regulation of other genes related to immune signaling, such as ubiquitination and phosphorylation, may also play important regulatory and antiviral functions, topics that should be further investigated.

On one hand, CypA increases the efficiency of transporting and utilizing immune cells between infected sites and other organs through the regulation of leukocyte chemotaxis, and decreases immunologic injury by reducing apoptosis. On the other hand, CypA enhances immune system development to repair impairments caused by the infection over time, while decreasing excessive immune provocations by down-regulating immune cell proliferation and activation. In addition, CypA promotes the recovery of damaged respiratory tissues by regulating the up-DEGs of respiratory tube development. These systematic antiviral functions might be accurately regulated by the immune signal pathways, including ubiquitination, phosphorylation, and other signaling pathways in hosts overexpressing CypA. Interestingly, the CypA-CMV mice were more resistant to disease than the CypA-SPC mice, and this was reflected in the MLD_50_ calculations during infection with A/WSN/33(H1N1). The over-expression of CypA from the CMV promoter all over the body of the host likely increased its disease resistance threshold against influenza virus infection.

This study is the first to investigate CypA antiviral effects *in vivo*, using influenza virus as an example. Due to the wide range of known roles for CypA in the regulation of immune responses as well as infectious diseases, the transgenic mouse model opens the door in confirming previous *in vitro* observations, as well as determining the exact roles and mechanisms of CypA in host immunity and pathogenesis of infectious diseases. In addition, the CypA+ transgenetic animal is undoubtedly paving the way for animal breeding with the design that simultaneously regulate immune responses and limit pathogen replication, which might be a better strategy than only target on virus itself as previous study^48^.

## Materials and Methods

### Construction and identification of transgenic mice over-expressing CypA

The cyclophilin A (CypA, IA) gene (GenBank No. AB451307) was cloned into the expression vectors pCMV-Myc and pCDNA3.1-SPC under control of the CMV and SPC promoters, respectively. The pDNA3.1-SPC construct was generated by our laboratory, in which the CMV promotor of pCDNA3.1 vector was replaced by the SPC promotor. The transgenic C57BL/6 mice were generated by the microinjection method as previously described^49^. The CMV or SPC promoters were designed to express the target gene over the entire body and specifically in the lungs of the transgenic mice, respectively. Genomic DNA from the transgenic mice was extracted from tail tissues for genotyping using PCR with the following primers: CMV-196S: 5′-GTCAATGGGTGGAGTATTTACG-3′; CMV-581A: 5′-GCTTATATAGACCTCCCACCGT-3′; SPC-768S: 5′-GCACTGAGACCTCCACATACTG-3′; and SPC-1124R: 5′-CCTTCCACCTCTCTGAATGC-3′. The desired 386-bp (CMV) and 357-bp (SPC) fragments of the promoters were amplified by 35 cycles of, 94 °C for 30 s, 55 °C for 30 s, and 72 °C for 30 s. The founders of the transgenic mice lines were also identified by western blotting with an antibody against CypA. The positive transgenic mice displaying more than 2-fold CypA overexpression in the lungs were selected for the founder lines.

### Determination of MLD_50_ in transgenic and wild type mice

Five 6- to 8-week-old female mice were anesthetized with tiletamine-zolazepam (Zoletil; Virbac; 25 μg/g) and inoculated intranasally (i.n.) with A/WSN/33(H1N1) virus at a dose of 1000, 2000, 4000, or 8000 PFU in 50 μL phosphate-buffered saline (PBS). Mock-infected control animals were inoculated i.n. with 50 μL PBS. The mice were monitored daily for a 14-day observation period, and the body weights were measured and expressed as a percentage of the initial value, calculated at day 0. Mice that lost ≥25% of its preinoculation body weight was pronounced dead and lose ≥30% of its preinoculation body weight were euthanized according to animal ethics guidelines. MLD_50_ values were calculated by the Reed and Muench method^50^ and expressed as the PFU value corresponding to 1 MLD_50_.

### Pathogenesis comparison in influenza-infected transgenic and wild type mice

Twenty-three wild type, CypA-CMV, and CypA-SPC mice were inoculated i.n. with A/WSN/33(H1N1) virus at a dose of 3000 PFU. The mice were monitored daily for general behavior and clinical signs, including food intake, body weight, and inactivity. Five stable mice in each group were segregated for weighing. Three mice from each group were euthanized at 3, 5, 7, 9, 11, and 14 days post-infection (d.p.i.), and their lungs were collected, weighed, and homogenized using a QIAGEN TissueLyser II machine (30 cycles/s, 4 min) in 1 mL of cold PBS under sterile conditions. Solid debris was pelleted by centrifugation at 5,000 × g for 10 min, and the homogenates were used for virus titrations in MDCK cells as previously published^51^. Their sera were used for cytokine detection by the cytometric bead array method using a Mouse Inflammation Kit (BD, USA). The lung index was calculated as the lung wet weight/body weight ×100. Additionally, a portion of each left lung lobe was fixed in 10% buffered formalin, embedded in paraffin, and 5-μm sections were stained with hematoxylin-eosin (H&E) and the monoclonal antibody against M1 protein for histopathological and immunohistochemical (IHC) analyses, respectively.

### RNA-seq analysis

The peritoneal macrophages of wild type and CypA-CMV mice were isolated from the peritoneal cavities and maintained in RPMI 1640 containing 10% fetal bovine serum at 37 °C until use, as described previously^52^. The cells were cultured in 24-well microplates, infected with A/WSN/1933(H1N1) at MOI = 2 or mock-infected with PBS, and harvested 8 h post-infection (h.p.i.) Total cellular RNA was extracted from the mixture of samples of three independent replications according to the manufacture’s protocol using Trizol (Invitrogen). All of the RNA Integrity Number (RIN) values were >7.0, and the 28S:18S rRNA ratios were >1.9, as confirmed using an Agilent Bioanalyzer. cDNA libraries were constructed from poly(A)-enriched RNA using Illumina kits and sequenced by 2*100 paired-end sequencing on an Illumina HiSeq 2000 instrument. The FASTQ read files for the four samples (A/WSN/1933(H1N1)- and mock-infected, wild type (WT) or CypA (IA)-overexpressing cells) were used for further data analysis. Data for gene counts were obtained using the Mayo Clinic pipeline and Burrows-Wheeler Alignment (BWA).

To analyze the Illumina reads, TopHat and Cufflinks were used to investigate the differential gene expression (DGE) profiles and changes in transcript abundance in the CypA over-expressing and wild type peritoneal macrophages with or without A/WSN/1933(H1N1) infection. Four files of transcriptome data from the IA-WSN, WT-WSN, IA-Mock (Mk), and WT-Mk groups were aligned to the UCSC Rhesus Macaque genome build in preparation for differential expression analysis. The four files were processed through Cufflinks to assemble the aligned RNA-seq reads into transcripts and estimate the abundances in FPKM of the paired–end reads. The Cufflinks q-value was the false discovery rate (FDR)-adjusted p-value of the uncorrected test statistic; the q-value used in this study was 0.05. The significance status was “yes” when p > q after Benjamini-Hochberg correction for multiple-testing. Cuffmerge was then used to create a single transcript dataset from the multiple reconstructions. Two runs were conducted using the IA-WSN *vs.* WT-WSN and the IA-Mk *vs.* WT-Mk datasets and the Cuffdiff program to test for differential expression and regulation among the two compared datasets. All significant hits were used to analyze the specific up- and down-regulation of gene expression of the A/WSN/1933(H1N1)-infected peritoneal macrophages from the CypA transgenic mice by Venn diagrams^53^. The gene functional classification of the associated up- and down-regulated genes were analyzed by DAVID Bioinformatics Resources^54^, and the Heat-map was analyzed using Cluster 3.0 and TreeView 1.6.0 software (Michael Eisen, Berkeley, CA).

### Ethics statement

The animal research was approved by the Chinese Academy of Sciences of Research Ethics Committee, under approval number PZIMCAS2013001, and complied with the Beijing Laboratory Animal Welfare and Ethical Guidelines of the Beijing Administration Committee of Laboratory Animals.

## Additional Information

**How to cite this article**: Li, J. *et al*. Cyclophilin A protects mice against infection by influenza A virus. *Sci. Rep.* **6**, 28978; doi: 10.1038/srep28978 (2016).

## Supplementary Material

Supplementary Information

## Figures and Tables

**Table 1:** MLD_50_ values of A/WSN/33(H1N1) influenza virus infecting wild-type and transgenic C57BL/6 mice.

Genotype	MLD_50_ (PFU)*	Fold increase (relative to C57BL/6)
C57BL/6	1561	1
CypA-SPC	3892	2.5
CypA-CMV	5907	3.8

^*^MLD_50_ (PFU): 50% median lethal dose (plaque forming units).
