# Supplementary material for: Cyclophilin A protects mice against infection by influenza A virus
Source: Sci Rep. 2016 Jun 29;6:28978. doi: 10.1038/srep28978 (PMC4926061; doi:10.1038/srep28978)
Supplement: Supplementary Information [file srep28978-s1.pdf]

Cyclophilin A protects mice against infection by influenza A virus

Jing Li[1], Can Chen[1], Gary Wong[1], Wei Dong[3], Weinan Zheng[1], Yun Li[1], Lei Sun[1], Lianfeng Zhang[3], George F. Gao[1,2], Yuhai Bi[1,2]*, and Wenjun Liu[1,2]*

1. CAS Key Laboratory of Pathogenic Microbiology and Immunology, Institute of Microbiology, Chinese Academy of Sciences, Beijing 100101, China.

2. Center for Influenza Research and Early-warning (CASCIRE), Chinese Academy of Sciences, Beijing 100101, China.

3. Key Laboratory of Human Disease Comparative Medicine, Ministry of Health, Institute of Laboratory Animal Science, Chinese Academy of Medical Sciences & Comparative Medical Center, Peking Union Medical College, Beijing, 100021 China,

* Corresponding authors: Yuhai Bi or Wenjun Liu

Email: Beeyh@im.ac.cn; Liuwj@im.ac.cn

Tel (+86) 10 64807497; Fax (+86) 10 64807503

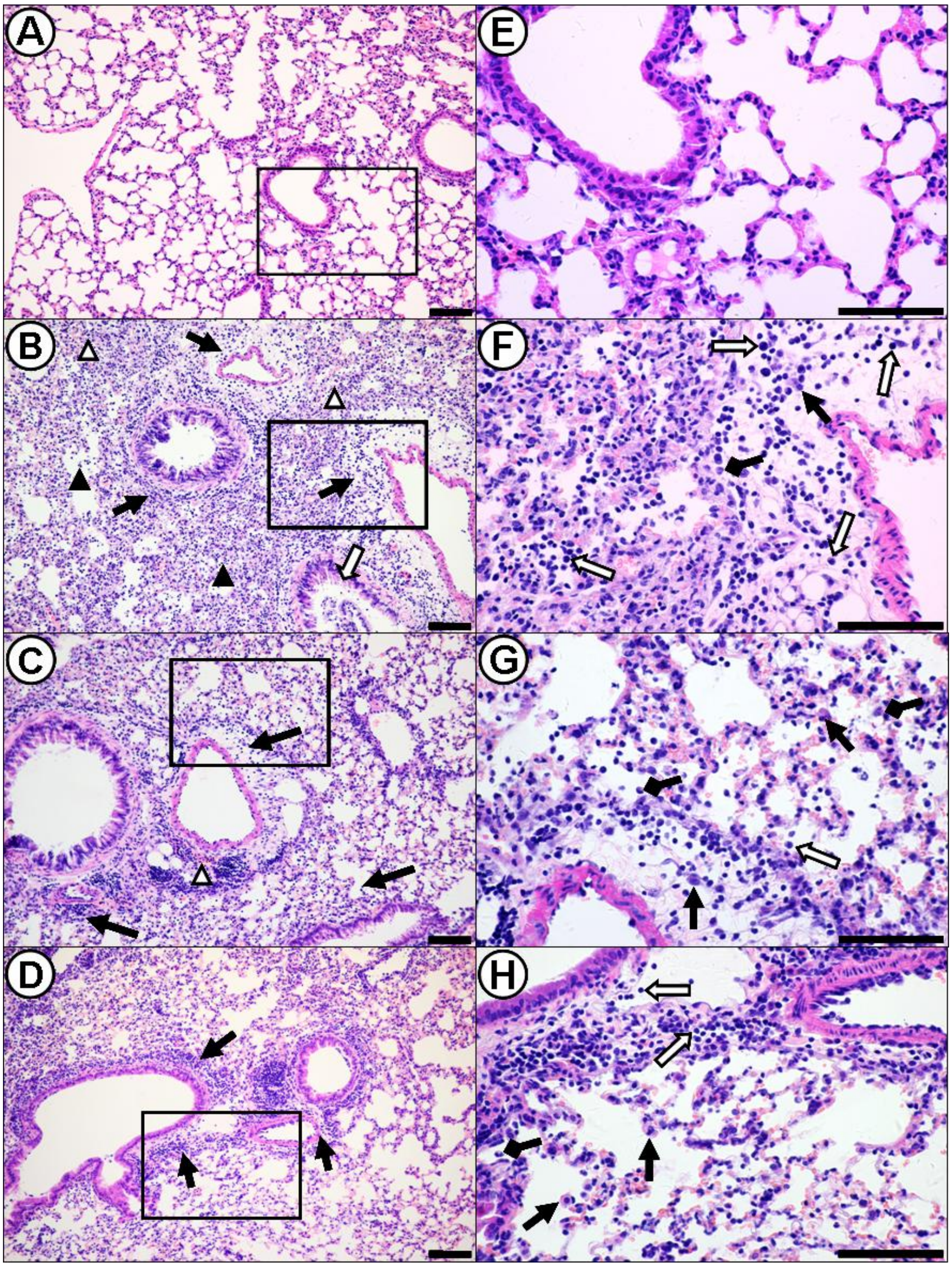

Figure S1. Hematoxylin and eosin (H&E)-stained lung sections of the infected mice at 7 d.p.i.

Lung histopathology sections (magnification, 200x) of mice were shown at 7 d.p.i. for the (A) PBS mock-infection control, (B) wild type, (C) CypA-SPC, and (D) CypA-CMV groups. (E to H) Enlargements (600x) for panels A to D, respectively.
